# Metabolic bulk volume from FDG PET as an independent predictor of progression-free survival in follicular lymphoma

**DOI:** 10.3389/fonc.2023.1283582

**Published:** 2023-11-03

**Authors:** Heejune So, Hyunjong Lee, Seung Hyup Hyun, Young Seok Cho, Seung Hwan Moon, Joon Young Choi, Kyung-Han Lee

**Affiliations:** ^1^ School of Medicine, University of Galway, Galway, Ireland; ^2^ Department of Nuclear Medicine, Samsung Medical Center, Sungkyunkwan University School of Medicine, Seoul, Republic of Korea

**Keywords:** follicular lymphoma, positron emission tomography (PET), computed tomography (CT), ^18^F-fluorodeoxyglucose (FDG), bulky, metabolic tumor volume

## Abstract

**Background:**

Total metabolic tumor volume (TMTV) in ^18^F-fluorodeoxyglucose (FDG) positron emission tomography (PET) predicts patient outcome in follicular lymphoma (FL); however, it requires laborious segmentation of all lesions. We investigated the prognostic value of the metabolic bulk volume (MBV) obtained from the single largest lesion.

**Methods:**

Pretreatment FDG PET/computed tomography (CT) scans of 201 patients were analyzed for TMTV and MBV using a 41% maximum standardized uptake value (SUVmax) threshold.

**Results:**

During a median follow-up of 3.2 years, 54 events, including 14 deaths, occurred. Optimal cut-offs were 121.1 cm^3^ for TMTV and 24.8 cm^3^ for MBV. Univariable predictors of progression-free survival (PFS) included a high Follicular Lymphoma International Prognostic Index 2 (FLIPI2) score, TMTV, and MBV. In the multivariable analysis, high TMTV and MBV were independent predictors of worse PFS (*P* =0.015 and 0.033). Furthermore, in a sub-group with FLIP2 scores of 0–2 (*n* = 132), high MBV could identify patients with worse PFS (*P* = 0.007).

**Conclusion:**

Readily measurable MBV is useful for stratifying risk in FL patients.

## Introduction

Follicular lymphoma (FL) is a heterogeneous disease with various clinical parameters that influence outcomes. The treatment choice for FL depends on the disease characteristics (1). Although rituximab has improved survival, many subjects treated with immunochemotherapy still show early progression or relapse (2). This underscores the importance of risk stratification in FL patients.

The Follicular Lymphoma International Prognostic Index 2 (FLIPI2) model was built to assess progression-free survival (PFS) in response to chemoimmunotherapy (3). ^18^F-fluorodeoxyglucose (FDG) positron emission tomography (PET) also provides valuable prognostic information in the rituximab era (4). The volumetric FDG parameter of total metabolic tumor volume (TMTV) strongly correlates with FL survival outcome (4). This finding was established by a pooled analysis of three prospective studies of FL patients at 36 PET centers, which demonstrated that baseline TMTV has the highest hazard ratio (HR) for survival (5).

However, the routine use of TMTV measurement is hampered by the time-consuming segmentation of all lesions in the body. This is further complicated by the heterogeneity of FDG uptake between lymphoma lesions (6), which hinders accurate TMTV measurement (7), particularly in the presence of small lesions with low FDG uptake (8).

Bulky lymphoma remains a simple but significant prognostic indicator that does not consider smaller lesions (9). This suggests the potential usefulness of metabolic bulk volume (MBV) as a prognostic parameter that is more easily measured. Our group and others recently demonstrated this finding in patients with diffuse large B-cell lymphoma (DLBCL) (10, 11). FL is fundamentally different from DLBCL in that it is generally incurable, with progression within the first two years of immunochemotherapy being a strong indicator of a poor outcome (12). In this study, we investigated the prognostic value of baseline MBV in a cohort of 201 FL patients treated with rituximab immunochemotherapy.

## Materials and methods

### Study population

Study candidates were pathology-confirmed FL patients who underwent FDG PET/CT imaging at our institution between 2012 and 2020. Inclusion criteria were adult patients (age > 18 years) of either gender or any Ann Arbor stage, who underwent baseline FDG PET/CT (n = 224). Exclusion criteria were patients whose first-line treatment regimen did not include rituximab (n = 20) or had PET/CT data errors (n = 3). Thus, a total of 201 FL patients were finally included for analysis. This retrospective study was approved by our institutional review board, and the requirement for informed consent was waived.

### FDG PET/CT imaging

The patients fasted for a minimum of 6 h to reach a blood glucose level < 150 mg and were injected with 5 MBq/kg of FDG. PET/CT was performed approximately 75 min later without intravenous or oral contrast. Images were acquired on a Discovery STE PET/CT scanner (GE Healthcare, Chicago, IL). Following continuous spiral CT with a 16-slice helical CT (140 keV; 30–170 mA), an emission scan was obtained from the head to the thigh for 2.5 min per frame. Attenuation-corrected images were used for a three-dimensional mode of reconstruction (3.9 × 3.9 × 3.3 mm) using an ordered-subset expectation maximization algorithm (20 subsets and two iterations).

### Review of PET/CT images and analysis of FDG uptake

PET, CT, and fusion PET/CT images were reviewed in axial, coronal, and sagittal planes on Advantage Workstation 4.4 (GE Healthcare). Quantification in each patient was performed while blinded to all clinical information, including treatment outcomes. Maximum tumor dimension (MTD) was defined as the largest lesion’s maximum transverse diameter on CT images. The SUVmax of the highest FDG uptake lesion was recorded.

MBV was defined as the largest MTV. To obtain MTV values, a cubical bounding volume of interest was first drawn around each target lesion with care not to include areas of high physiological uptake (brain, heart, liver, kidney, or bladder). A 41% threshold of the local tumor SUVmax was then applied for lesion segmentation, and any region of physiological uptake was manually excluded. The MTV of a lesion was measured as the sum of all voxels with FDG uptake exceeding the threshold of the local SUVmax. The largest MTV obtained was recorded as the MBV. The TMTV was calculated by summing the MTVs of all VOIs.

### Medical record review and follow-up

Clinical information was obtained from our institutional information system. Medical records were reviewed for clinical characteristics including age and sex. Baseline laboratory data within one week of PET/CT included serum lactate dehydrogenase, hemoglobin, and white blood cell and platelet counts. FLIPI2 risk scores were calculated from these data.

Patients underwent interim and end-of-treatment follow-up PET/CT imaging. Disease progression was defined by PET/CT and clinical evidence of lymphoma progression. Disease relapse after treatment was defined as clinical or imaging evidence of recurrence during follow-up.

### Statistical analyses

Continuous variables were first evaluated for normality using Kolmogorov-Smirnov tests. The results showed that age and hemoglobulin levels followed a normal distribution while other variables did not. Hence, comparisons between MBV groups were performed by two-sided Student’s t-tests for age and hemoglobulin level while nonparametric two-sided Mann-Whitney tests were used for the remaining clinical variables. Categorical and discrete data were compared by Pearson’s chi-square tests. *P* values < 0.05 were considered significant. The primary endpoint for survival analyses was overall survival (OS) or PFS. OS was defined as the time from baseline PET/CT (one to two days before the start of chemotherapy) to the day of death from any cause. PFS was defined as the time from baseline PET/CT to the day of primary progression, recurrence, or death from any cause. Patients alive at the last follow-up were counted as censored observations. ROC curve analysis identified the optimal cut-off values of MTD, TMTV, and MBV for event prediction.

Survival curves were obtained from Kaplan–Meier estimates and compared using the log-rank test. Univariable and multivariable Cox proportional hazards regression analysis was performed using the Statistical Package for the Social Sciences (SPSS) version 23.0 (IBM Corp., Armonk, NY). Multivariable analysis was performed including variables whose association with survival on univariable Cox analysis was statistically significant (P <0.05) or showed a trend towards significance (P <0.10). The selection was done to avoid overfitting, given the limited numbers of events. Variables with a level of significance <0.10 were included to reduce omitted-variable bias.

## Results

### Clinical and pathological features

The mean subject age was 52.1 ± 12.1 years, and 45.8% were male. The Ann Arbor Stage was I in 14 cases, II in 25, III in 57, and IV in 105. The FILPI2 risk score was ≥3 in 69 (34.3%) subjects. The mean blood levels were 13.0 ± 1.7 g/dL for hemoglobin, 2.4 ± 1.5 mg/L for β2 microglobulin, and 350.4 ± 296.3 IU/L for lactate dehydrogenase (**Table 1**). The patients received rituximab alone (3.0%); rituximab combined with cyclophosphamide, vincristine, doxorubicin, and prednisolone (R-CHOP; 14.4%); rituximab combined with cyclophosphamide, vincristine, and prednisolone R-CVP, 27.4%); or bendamustine with rituximab (BR; 55.2%).

**Table 1:** Clinical characteristics of 201 FL patients according to MBV.

Clinical characteristic	Number of patients (%)			*P* value
	Total	Low MBV	High MBV	
Male gender	92 (45.8%)	38 (43.2%)	54 (47.8%)	0.516
Age > 60	54 (26.9%)	26 (29.6%)	28 (24.8%)	0.572
Ann Arbor stage III–IV	162 (80.6%)	67 (76.1%)	95 (84.1%)	0.158
Hemoglobulin < 12 g/dL	46 (22.9%)	16 (18.2%)	30 (26.6%)	0.161
LDH > 225 IU/L	154 (76.6%)	68 (77.3%)	86 (76.1%)	0.846
β2 microglobulin ≥ 2.0 IU/L	149 (74.1%)	58 (65.9%)	91 (80.5%)	0.075
Number of nodal sites ≥ 5	93 (46.3%)	34 (38.6%)	59 (52.2%)	0.055
Bone marrow involvement	116 (57.7%)	45 (51.1%)	71 (62.8%)	0.096
MTD > 60 mm	77 (38.3%)	3 (3.4%)	74 (65.5%)	<0.001
FLIPI2 score ≥ 3	69 (34.3%)	13 (14.8%)	56 (49.6%)	<0.001.
SUVmax > 4.0	142 (70.7%)	65 (73.9%)	77 (68.1%)	0.377
Treatment regimen				0.079
- Rituximab	6 (3.0%)	5 (5.7%)	(0.9%)	
- R-CHOP	29 (14.4%)	10 (11.4%)	19 (16.8%)	
- R-CVP	55 (27.4%)	20 (22.7%)	35 (31.0%)	
- BR	111 (55.2%)	53 (60.2%)	58 (51.3%)	
Mean age (years)	52.1 ± 12.1	53.0 ± 12.7	51.3 ± 11.6	0.324
Mean hemoglobulin (g/dL)	13.0 ± 1.7	13.3 ± 1.6	12.8 ± 1.7	0.061
Mean LDH (IU/L)	350.4 ± 296.3	325.6 ± 180.9	368.4 ± 357.6	0.279
Mean β2 microglobulin (IU/L)	2.4 ± 1.5	1.9 ± 0.9	2.7 ± 1.7	0.001†
Mean SUVmax	5.9 ± 3.7	5.6 ± 2.4	6.2 ± 4.4	0.306

MBV, metabolic bulky volume; high MBV, > 24.8 cm^3^; LDH, lactate dehydrogenase; MTD, maximum transverse diameter; FLIPI2, FL International Prognostic Index 2; SUVmax, maximum standard uptake value. R-CHOP, rituximab + cyclophosphamide + vincristine + doxorubicin + prednisolone; R-CVP, rituximab + cyclophosphamide + vincristine + prednisolone; BR, bendamustine + rituximab. †, by the nonparametric Mann-Whitney U test.

### Quantitative assessment of FDG uptake on PET/CT

The largest lymphoma lesion had a maximum tumor dimension (MTD) of 60.9 ± 42.0 mm, and the mean maximum standard uptake value (SUVmax) for the lesion with the highest FDG uptake was 5.9 ± 3.7. The mean TMTV was 311.4 ± 430.5 cm^3^ and the mean MBV was 126.2 ± 252.9 cm^3^.

### Patients with high or low MBV

Receiver operating characteristic (ROC) curve analyses showed that the optimal cut-offs for predicting events were 60 mm for MTD, 121.1 cm^3^ for TMTV, and 24.8 cm^3^ for MBV. The high-MBV group (*n* = 113) was more likely to have high MTD (*P* <0.001), increased β2 microglobulin (*P* <0.05), and high FILPI-2 risk scores (*P* <0.001), while other variables did not differ (**Table 1**). The immunochemotherapy regimen also did not significantly differ between low and high MBV groups (*P* = 0.08). Nonparametric tests showed significantly greater β2 microglobulin levels for the high MBV group compared to the low MBV group (*P* = 0.001).

### Survival and Kaplan–Meier analyses

During a median follow-up of 3.17 years (range = 0.04–9.67 years), events including progression, recurrence, and death occurred in 54 patients. Any other cause of death occurred in 14 patients at a median of 2.1 years.

**Figure 1:**
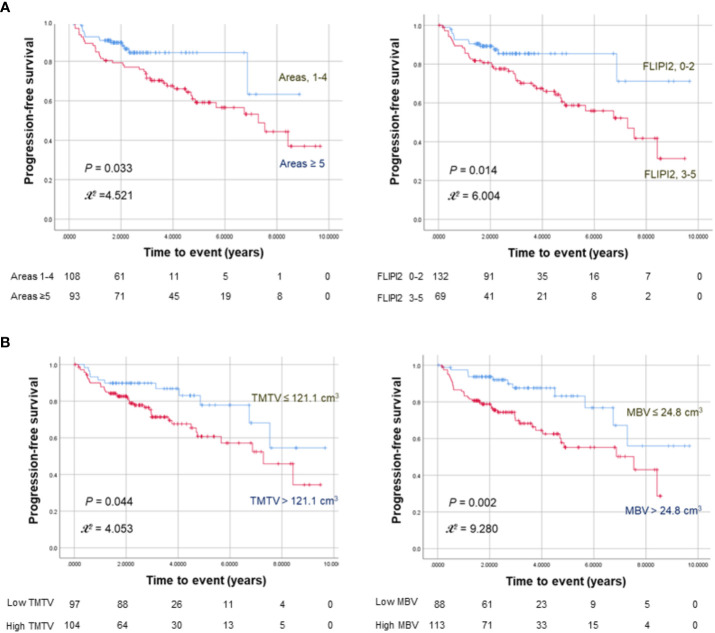
Kaplan–Meier curves for progression-free survival in 201 FL patients stratified by nodal lesion number or FLIPI2 risk score **(A)** and TMTV or MBV **(B)**. The numbers at risk for each group are shown as a table under the curves.

Kaplan–Meier survival analyses with log-rank tests revealed significantly worse PFS for ≥ 5 nodal lesions (*P* = 0.033), FLIPI2 score ≥ 3 (*P* = 0.017), high TMTV (*P* = 0.044), and high MBV (*P* = 0.002; **Figure 1**). OS was significantly worse for age > 60 years (*P* = 0.004).

### Univariable and multivariable predictors of survival

Cox univariable analysis showed that ≥ 5 nodal lesions (HR = 1.943; 95% CI = 1.04–3.62; *P* = 0.036), FLIPI2 score ≥ 3 (HR = 2.171; 95% CI = 1.15–4.10; *P* = 0.017), high TMTV (HR = 1.953; 95% CI = 1.01–3.79; *P* = 0.048), and high MBV (HR = 2.614; 95% CI = 1.38–4.97; *P* = 0.003) were significant predictors of worse PFS (**Table 2**). High MTD was not a significant univariable predictor of worse PFS (HR: 1.476; 95% CI: 0.86-2.53; *P* = 0.156).

**Table 2:** Univariable and multivariable analyses for progression-free survival.

Clinical variables	Events	HR	95% CI	*P*
Univariable analysis				
Age > 60 years	17/54	1.181	0.68–2.12	0.577
Male gender	30/92	1.389	0.81–2.37	0.229
Ann Arbor Stage III–IV	45/162	1.111	0.56–2.18	0.764
Hemoglobulin < 12 g/dL	14/46	1.229	0.66–2.30	0.519
Lactate dehydrogenase > 225 IU/L	51/154	2.484	0.88–6.99	0.085
β2 microglobulin ≥ 2.0	43/149	1.417	0.74–2.70	0.289
Number of nodal sites ≥ 5	40/93	1.943	1.04–3.62	0.036
Bone marrow involvement	29/116	1.301	0.73–2.31	0.370
MTD > 60 mm	24/77	1.426	0.86-2.53	0.156
FLIPI2 score ≥ 3	23/69	2.171	1.15–4.10	0.017
SUVmax > 4.0	44/142	1.476	0.86–2.53	0.672
TMTV > 121.1 cm^3^	34/109	1.953	1.01–3.79	0.048
MBV > 24.8 cm^3^	39/113	2.614	1.38–4.97	0.003
Multivariable analysis including TMTV				
TMTV > 121.1 cm^3^		3.027	1.25–7.40	0.015
Lactate dehydrogenase > 225 IU/L		1.420	0.58–3.49	0.147
Number of nodal sites ≥ 5		1.382	0.70–2.72	0.348
FLIPI2 score ≥ 3		0.193	0.57–1.75	0.980
Multivariable analysis including MBV				
MBV > 24.8 cm^3^		2.407	1.08–5.39	0.033
Lactate dehydrogenase > 225 IU/L		1.384	0.57–3.39	0.426
Number of nodal sites ≥ 5		1.696	0.89–3.23	0.108
FLIPI2 score ≥ 3		0.925	0.52–1.66	0.793

HR, hazard ratio; CI, confidence interval; MTD, maximum transverse diameter; FLIPI2, FL International Prognostic Index 2; SUVmax, maximum standard uptake value; TMTV, total metabolic tumor volume; MBV, metabolic bulky volume. Total events = 54/201 (26.9%).

To test the robustness of its prognostic value, we repeated Cox univariate analyses according to MBV groups using a range of cutoff values (10% and 20% greater or smaller than 24.8 cm^3^). The results showed significant associations with worse PSF for MBV > 29.8 cm^3^ (HR = 1.79; *P* = 0.044), MBV > 27.3 cm^3^ (HR = 1.87; *P* = 0.033), MBV > 22.3 cm^3^ (HR = 2.04; *P* = 0.022), and MBV > 19.8 cm^3^ (HR = 1.91; *P* = 0.037).

Multivariable Cox proportional hazards analysis revealed that a high TMTV (HR = 3.027; 95% CI = 1.25–7.40; *P* = 0.015) was the only significant independent predictor of worse PFS when it was included in the model. Likewise, a high MBV (HR = 2.407; 95% CI = 1.08–5.39; *P* = 0.033) was the only significant independent predictor when it was included (**Table 2**).

Older age was the only significant univariate predictor for worse OS (HR = 4.24; 95% CI = 1.46–12.3; *P* = 0.008). On multivariable analyses, age was the only independent predictor for worse OS (HR = 4.053; 95% CI = 1.40–11.7; *P* = 0.010; **Supplementary Table 1**).

### Combining MBV and FLIPI2 scores for prediction of PFS

**Figure 2:**
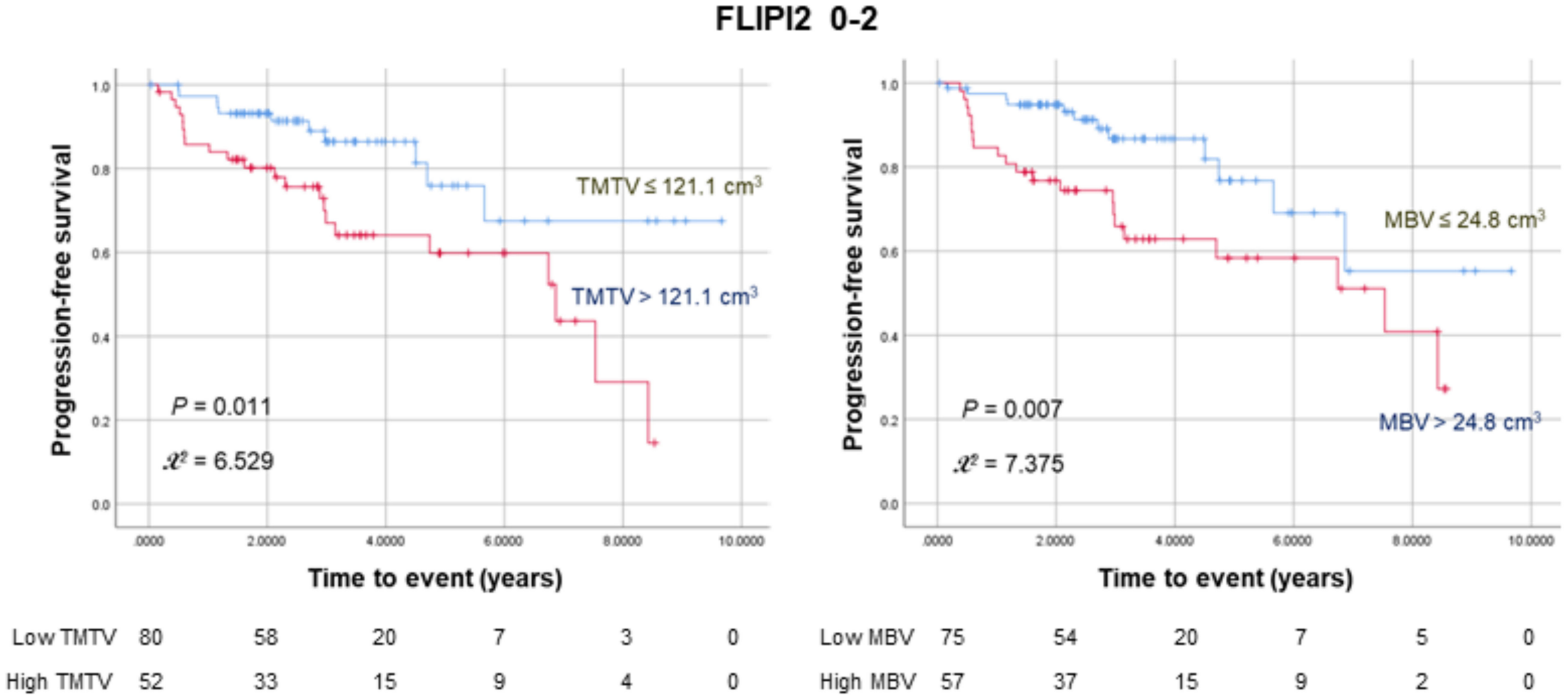
Kaplan–Meier curves for progression-free survival in 132 patients with low to intermediate FLIPI2 risk scores (0–2) stratified according to TMTV (left) or MBV (right). The numbers at risk for each group are shown as a table under the curves.

Among a subgroup of 132 patients with low to intermediate FLIP2 scores (0–2), 52 (39.4%) had a high TMTV, of whom 21 experienced events. Similarly, 57 (43.2%) had a high MBV, of whom 22 experienced events. Kaplan–Meier analysis in this subgroup revealed that both a high TMTV (*P* = 0.011) and MBV (*P* = 0.007) were associated with significantly worse PFS (**Figure 2**). In contrast, the high FLIPI2 group (3-5) showed no significant influence of MBV and TMTV on PFS (**Supplementary Figure 1A**).

We also tested the potential effects of β2 microglobulin level, which was increased in the high MBV group, but found no significant association with PFS in either low to intermediate or high FLIPI2 groups (**Supplementary Figure 1A**).

## Discussion

In our study of a homogeneous cohort of FL patients treated with rituximab, PFS was significantly associated with TMTV and MBV, along with the FLIPI2 score and number of nodal sites. Importantly, TMTV and MBV were the only independent predictors of worse PFS in multivariable analyses.

TMTV has been shown to offer prognostic value in many lymphoma types (4, 13). Meignan et al. extended this finding to FL by establishing a strong independent association of baseline TMTV with survival (5), and our results corroborate this link. MTV is generally measured using a fixed percentage SUVmax threshold. However, we previously reported that bulky DLBCL lesions with high FDG uptake were better delineated by a 30% SUVmax threshold (10), and bulky FL lesions in the present study were adequately delineated by a 41% SUVmax threshold, as suggested by the European Association of Nuclear Medicine guidelines (14). The optimal TMTV cut-off for events in our results was 121.1 cm^3^, which is significantly smaller than the 510 cm^3^ found by Meignan et al., who also used a 41% SUVmax threshold. This result could have been due to the lower total tumor burden (80% versus 92% stage III–IV disease) as well as the differences in treatments. PET scanner type and acquisition protocols are further sources of SUVmax variability that can influence TMTV results (7). The optimal TMTV cut-off for survival in DLBCL patients was reported to have a similarly wide range between 150 and 550 cm^3^ (15).

Compared with TMTV measured from all lesions, which is time-intensive and more prone to variability, MBV is readily obtained from the single largest metabolic lesion. In DLBCL, Delaby et al. (11) and our group (10) previously showed that a high MBV is an independent predictor of poor survival. The present study extended this association to FL, with TMTV and MBV as the only independent predictors of PFS.

In clinical practice, MTV is more likely to be utilized in combination with clinical risk scores. In Meignan’s study, combining TMTV with FLIPI2 scores could sub-stratify patient outcomes (5). We further explored whether MBV could sub-stratify survival in 65.7% of our subjects with low to intermediate FLIPI2 scores. As a result, among patients with a FLIP2 score of 0–2, 39.4% with a high TMTV and 43.2% with a high MBV had significantly worse PFS compared with their counterparts. These subjects had a median PFS of 4.74–5.67 years, suggesting that they may not be appropriately considered to have indolent disease.

A limitation of our study is that the cutoffs selected by ROC curve analyses are potentially data-driven. However, the fact that MBV groups categorized using a range of cutoff values above and below the value selected by ROC curve analyses remained significantly associated with PFS supports the prognostic value of this FDG parameter.

Given the limitations of routine TMTV measurements, our results support the use of MBV as a convenient and useful volumetric prognostic indicator. They further indicate that MBV helps sub-stratify the risk of early progression in patients with low to intermediate clinical risk. However, the encouraging results of this retrospective study need to be confirmed by future prospective investigations.

## Conclusion

Readily obtainable MBV is a significant independent predictor of PSF in FL and can sub-stratify the risk of early progression in patients with low to intermediate clinical risk.

## Data availability statement

The original contributions presented in the study are included in the article/**Supplementary Material**. Further inquiries can be directed to the corresponding author.

## Ethics statement

The studies involving humans were approved by Samsung Medical Center Institutional Review Board. The studies were conducted in accordance with the local legislation and institutional requirements. The ethics committee/institutional review board waived the requirement of written informed consent for participation from the participants or the participants’ legal guardians/next of kin because the retrospective study was based on image analysis and medical chart review with no potential adverse effects to the study subjects.

## Author contributions

HS: Data curation, Formal Analysis, Methodology, Writing – original draft, Writing – review & editing. HL: Formal Analysis, Writing – review & editing. SH: Methodology, Writing – review & editing. YC: Investigation, Writing – review & editing. SM: Formal Analysis, Methodology, Writing – review & editing. JC: Project administration, Writing – review & editing. K-HL: Conceptualization, Funding acquisition, Project administration, Supervision, Writing – original draft, Writing – review & editing.
