# Supplementary material for: Metabolic bulk volume from FDG PET as an independent predictor of progression-free survival in follicular lymphoma
Source: Front Oncol. 2023 Nov 3;13:1283582. doi: 10.3389/fonc.2023.1283582 (PMC10655116; doi:10.3389/fonc.2023.1283582)
Supplement: Supplementary file 1 [file Table_1.pdf]

SUPPLEMENTARY TABLE 1. Univariable analyses for overall survival.

Clinical variables	Deaths	HR	95% CI	*P*
**Univariable analysis**				
Age > 60 years	8/54	4.242	1.46–12.3	0.008
Male gender	8/92	1.561	0.54–4.50	0.410
Ann Arbor Stage III–IV	10/162	0.559	0.18–1.78	0.326
Hemoglobulin < 12 g/dL	5/46	2.095	0.70–6.28	0.186
Lactate dehydrogenase > 225 IU/L	13/154	1.018	0.21–4.84	0.928
β2 microglobulin ≥ 2.0	10/149	1.063	0.33–3.40	0.918
Number of nodal sites ≥ 5	12/93	3.948	0.84–18.4	0.081
Bone marrow involvement	6/116	0.818	0.26–2.55	0.729
MTD > 60 mm	4/77	0.707	0.22-2.28	0.562
FLIPI2 score ≥ 3	6/69	1.545	0.54–4.46	0.421
SUVmax > 4.0	12/142	2.675	0.60–11.9	0.198
TMTV > 121.1 cm^3	5/109	1.554	0.52–4.64	0.558
MBV > 24.8 cm^3	8/113	0.971	0.37–2.80	0.956
**Multivariable analysis**				
Age > 60 years		4.053	1.40–11.7	0.010
Number of nodal sites ≥ 5		3.734	0.79–17.6	0.095

HR, hazard ratio; CI, confidence interval; MTD, maximum transverse diameter; FLIPI2, FL International Prognostic Index 2; SUVmax, maximum standard uptake value; TMTV, total metabolic tumor volume; MBV, metabolic bulky volume. Total deaths = 14/201 (7.0%).
