# Supplementary material for: Metabolic bulk volume from FDG PET as an independent predictor of progression-free survival in follicular lymphoma
Source: Front Oncol. 2023 Nov 3;13:1283582. doi: 10.3389/fonc.2023.1283582 (PMC10655116; doi:10.3389/fonc.2023.1283582)
Supplement: Supplementary file 2 [file Image_1.pdf]

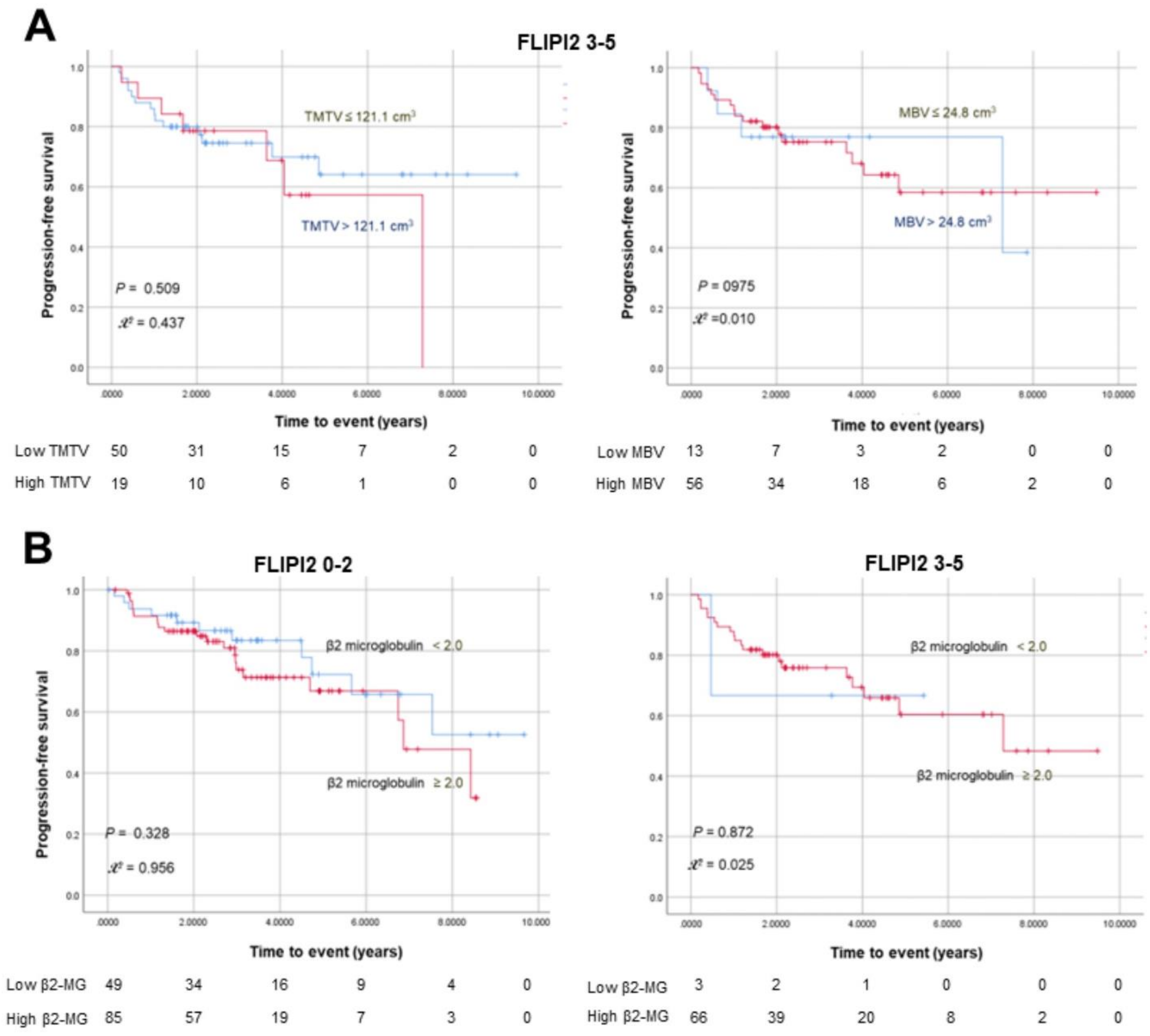

Supplementary Figure 1. (A) Kaplan–Meier curves for progression-free survival in 69 patients with high FLIPI2 risk scores (3–5) stratified according to TMTV (left) or MBV (right). (B) Kaplan–Meier curves for progression-free survival in 132 patients with low to intermediate FLIPI2 risk scores (0–2; left) and 69 patients with high FLIPI2 risk scores (3–5; right) stratified according to β2 microglobulin level. The numbers at risk for each group are shown as a table under the curves.
